# Novel and potent antimicrobial effects of caspofungin on drug-resistant *Candida* and bacteria

**DOI:** 10.1038/s41598-020-74749-8

**Published:** 2020-10-20

**Authors:** Makoto Sumiyoshi, Taiga Miyazaki, Juliann Nzembi Makau, Satoshi Mizuta, Yoshimasa Tanaka, Takeshi Ishikawa, Koichi Makimura, Tatsuro Hirayama, Takahiro Takazono, Tomomi Saijo, Hiroyuki Yamaguchi, Shintaro Shimamura, Kazuko Yamamoto, Yoshifumi Imamura, Noriho Sakamoto, Yasushi Obase, Koichi Izumikawa, Katsunori Yanagihara, Shigeru Kohno, Hiroshi Mukae

**Affiliations:** 1 Department of Respiratory Medicine, Nagasaki University Graduate School of Biomedical Sciences, 1-12-4 Sakamoto, Nagasaki, 852-8523 Japan; 2 Department of Respiratory Medicine, Nagasaki University Hospital, 1-7-1 Sakamoto, Nagasaki, 852-8501 Japan; 3 Department of Infectious Diseases, Nagasaki University Graduate School of Biomedical Sciences, 1-7-1 Sakamoto, Nagasaki, 852-8501 Japan; 4 Department of Molecular Microbiology and Immunology, Nagasaki University Graduate School of Biomedical Sciences, 1-12-4 Sakamoto, Nagasaki, 852-8523 Japan; 5 Center for Bioinformatics and Molecular Medicine, Nagasaki University Graduate School of Biomedical Sciences, 1-12-4 Sakamoto, Nagasaki, 852-8523 Japan; 6 Center for Medical Innovation, Nagasaki University, 1-7-1 Sakamoto, Nagasaki, 852-8588 Japan; 7 Department of Chemistry, Biotechnology, and Chemical Engineering, Graduate School of Science and Engineering, Kagoshima University, 1-21-40 Korimoto, Kagoshima, 890-0065 Japan; 8 Medical Mycology, Graduate School of Medicine, Teikyo University, 2-11-1 Kaga, Itabashi-ku, Tokyo, 173-8605 Japan; 9 Department of Laboratory Medicine, Nagasaki University Hospital, 1-7-1 Sakamoto, Nagasaki, 852-8501 Japan

**Keywords:** Antifungal agents, Biofilms

## Abstract

Echinocandins, including caspofungin, micafungin, and anidulafungin, are first-line antifungal agents for the treatment of invasive candidiasis. They exhibit fungicidal activity by inhibiting the synthesis of β-1,3-d-glucan, an essential component of the fungal cell wall. However, they are active only against proliferating fungal cells and unable to completely eradicate fungal cells even after a 24 h drug exposure in standard time-kill assays. Surprisingly, we found that caspofungin, when dissolved in low ionic solutions, had rapid and potent antimicrobial activities against multidrug-resistant (MDR) *Candida* and bacteria cells even in non-growth conditions. This effect was not observed in 0.9% NaCl or other ion-containing solutions and was not exerted by other echinocandins. Furthermore, caspofungin dissolved in low ionic solutions drastically reduced mature biofilm cells of MDR *Candida auris* in only 5 min, as well as *Candida*-bacterial polymicrobial biofilms in a catheter-lock therapy model. Caspofungin displayed ion concentration-dependent conformational changes and intracellular accumulation with increased reactive oxygen species production, indicating a novel mechanism of action in low ionic conditions. Importantly, caspofungin dissolved in 5% glucose water did not exhibit increased toxicity to human cells. This study facilitates the development of new therapeutic strategies in the management of catheter-related biofilm infections.

## Introduction

Approximately 750,000 cases of invasive candidiasis occur annually according to global estimates, and mortality is approximately 40%, even in patients receiving antifungal treatment^1,2^. Echinocandins, including caspofungin, micafungin, and anidulafungin, have been widely used as first-line antifungal agents for the treatment of invasive candidiasis. These compounds inhibit β-1,3-d-glucan synthesis by targeting *FKS* gene products and exhibit fungicidal activity against *Candida* spp. in vitro^3,4^. However, the development of echinocandin-resistance due to hotspot mutations in *FKS* genes and the emergence of multidrug-resistant strains in *Candida glabrata* and *Candida auris* have recently become a major global concern^5–9^. Furthermore, biofilms produced by *Candida* spp. have increased antifungal resistance and tolerance, complicating the treatment of patients with medical implants such as central venous catheters^10,11^. Catheter removal is frequently effective against catheter-related infections, but immediate catheter removal or replacement is not always feasible due to multiple factors including patient conditions and comorbidities. Some studies reported that amphotericin B, echinocandins, or a combination of these drugs has antibiofilm activity, but the efficacies of antifungal lock therapy using these agents have not yet progressed to a practical level for clinical use^12–14^. The need for novel therapeutic strategies against biofilm-producing *Candida* spp. is increasing, but development of new antifungal drugs has decelerated. Therefore, this study focused on developing a rapid and effective therapy against drug-resistant and biofilm-forming *Candida* spp. using existing antifungal agents.

During our preliminary study exploring echinocandin-resistant mechanisms in *C. glabrata* under various environmental conditions, we found that caspofungin had potent antimicrobial activities under low ionic conditions, not only against *Candida* spp., but also against drug-resistant bacteria such as methicillin-resistant *Staphylococcus aureus* and multidrug-resistant *Pseudomonas aeruginosa*. Here, we investigated the detailed conditions regulating this unique antimicrobial capability of caspofungin and its underlying mechanisms in pathogenic organisms.

## Results

### Susceptibility studies

Antifungal minimum inhibitory concentrations (MICs) and sessile MICs (SMICs) for *Candida* cells are shown in Supplementary Tables S1 and S2, respectively. *C. glabrata* and *C. albicans* planktonic cells were susceptible to antifungals examined, while both *C. auris* planktonic and biofilm cells were multidrug-resistant. Paradoxical effect was observed with caspofungin and micafungin above the drug concentrations of 0.125 mg/L and 0.06 mg/L, respectively, at 24 h of drug exposure against *C. albicans* biofilm cells (Supplementary Fig. S1).

### Potent antimicrobial activity of caspofungin in low ionic solutions

**Figure 1:**
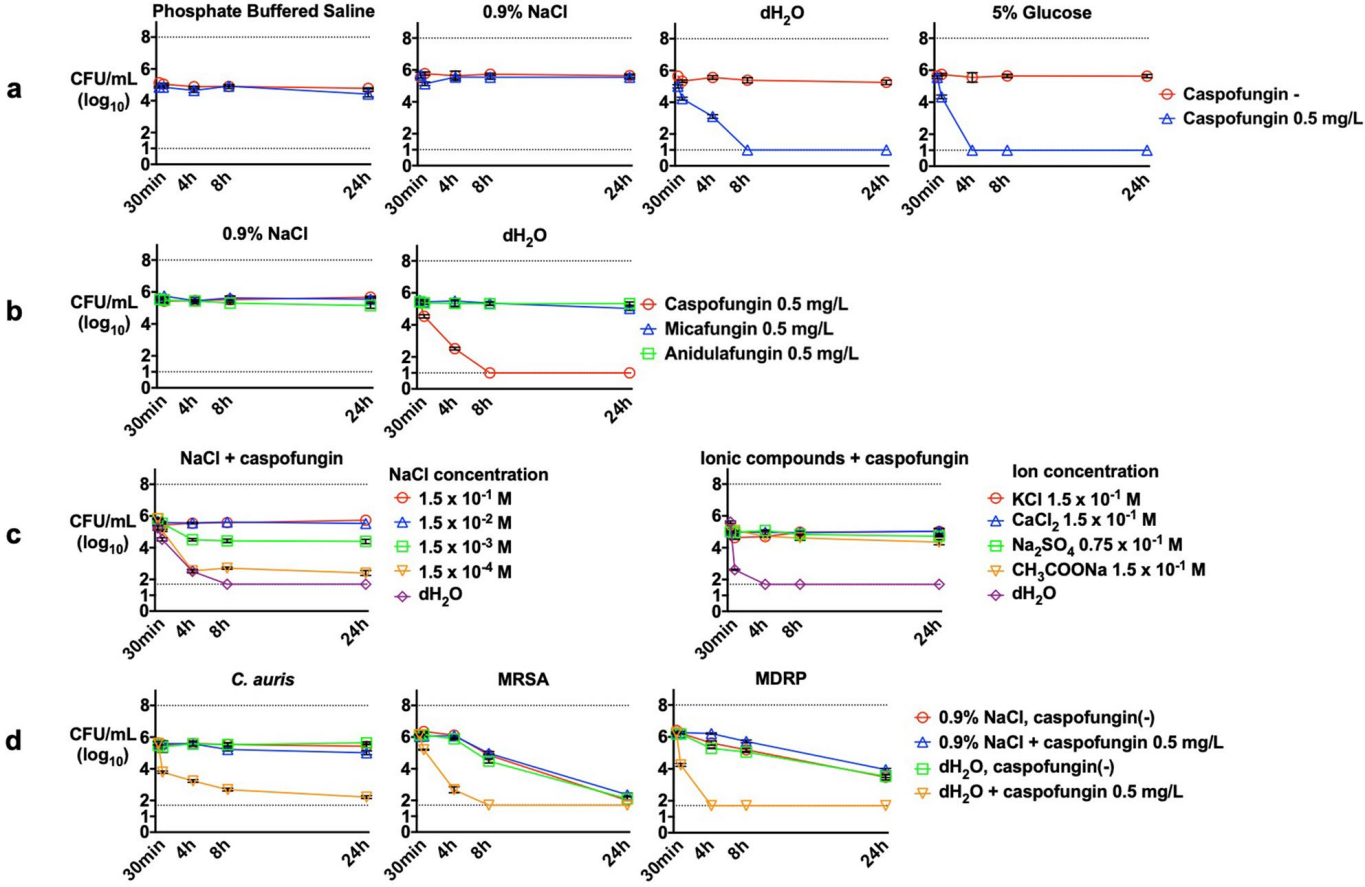
Time-kill assay of *Candida* and bacterial cells using echinocandin drugs under various conditions. Antifungal effects of (**a**) caspofungin under non-growth conditions, (**b**) caspofungin, micafungin, and anidulafungin under non-growth conditions, (**c**) caspofungin in the presence of different salt concentrations, and (**d**) caspofungin against *C. auris*, MRSA, and MDRP. Each data point represents the mean (± SD). Broken lines represent the limits of quantification at the upper (10^8^ CFU/mL) and lower limits (50 CFU/mL).

We evaluated the effect of caspofungin on *C. glabrata* viability in various media and solutions using time-kill assay. Caspofungin at 0.5 mg/L showed stronger antifungal effects in nutrition-rich yeast-peptone-dextrose (YPD) medium than in less nutritious media, such as minimal medium wherein only fungistatic effects were observed (Supplementary Fig. S2a). Caspofungin had no antifungal activity in phosphate buffered saline (PBS) or 0.9% NaCl, because *C. glabrata* cells were not able to proliferate in these conditions (Fig. 1a). However, caspofungin treatment resulted in an immediate 10‒100-fold decrease in cell count after 30 min and a further 10,000-fold decrease in 4‒8 h in 5% glucose water and distilled water (dH_2_O), despite the non-growth conditions. This potent fungicidal effect was exerted by caspofungin but not by other echinocandins (Fig. 1b).

The fungicidal activity of caspofungin was diminished by the presence of NaCl in a concentration-dependent manner and was also inhibited by other ionic compounds including KCl, CaCl_2_, CH_3_COONa, and Na_2_SO_4_ (Fig. 1c). When caspofungin was dissolved in dH_2_O containing NaCl at a concentration of 1.5 × 10^−4^ M or higher, the fungicidal activity of caspofungin decreased. However, the caspofungin activity was unaffected by osmolarity, as confirmed by using high sorbitol and glucose concentrations (Supplementary Fig. S2b), or by pH, which was confirmed by measuring the pH after caspofungin or micafungin addition (Supplementary Fig. S2c). We observed similar results for *C. glabrata* CGL305 and *C. albicans* where caspofungin exerted a potent fungicidal effect under low ionic solution, although higher concentrations of caspofungin were needed (Supplementary Fig. S2d). Surprisingly, this effect was further observed in *C. auris*, methicillin-resistant *Staphylococcus aureus* (MRSA), and multidrug-resistant *Pseudomonas aeruginosa* (MDRP) strains (Fig. 1d).

### Rapid reduction of biofilm cells via caspofungin dissolved in 5% glucose water

**Figure 2:**
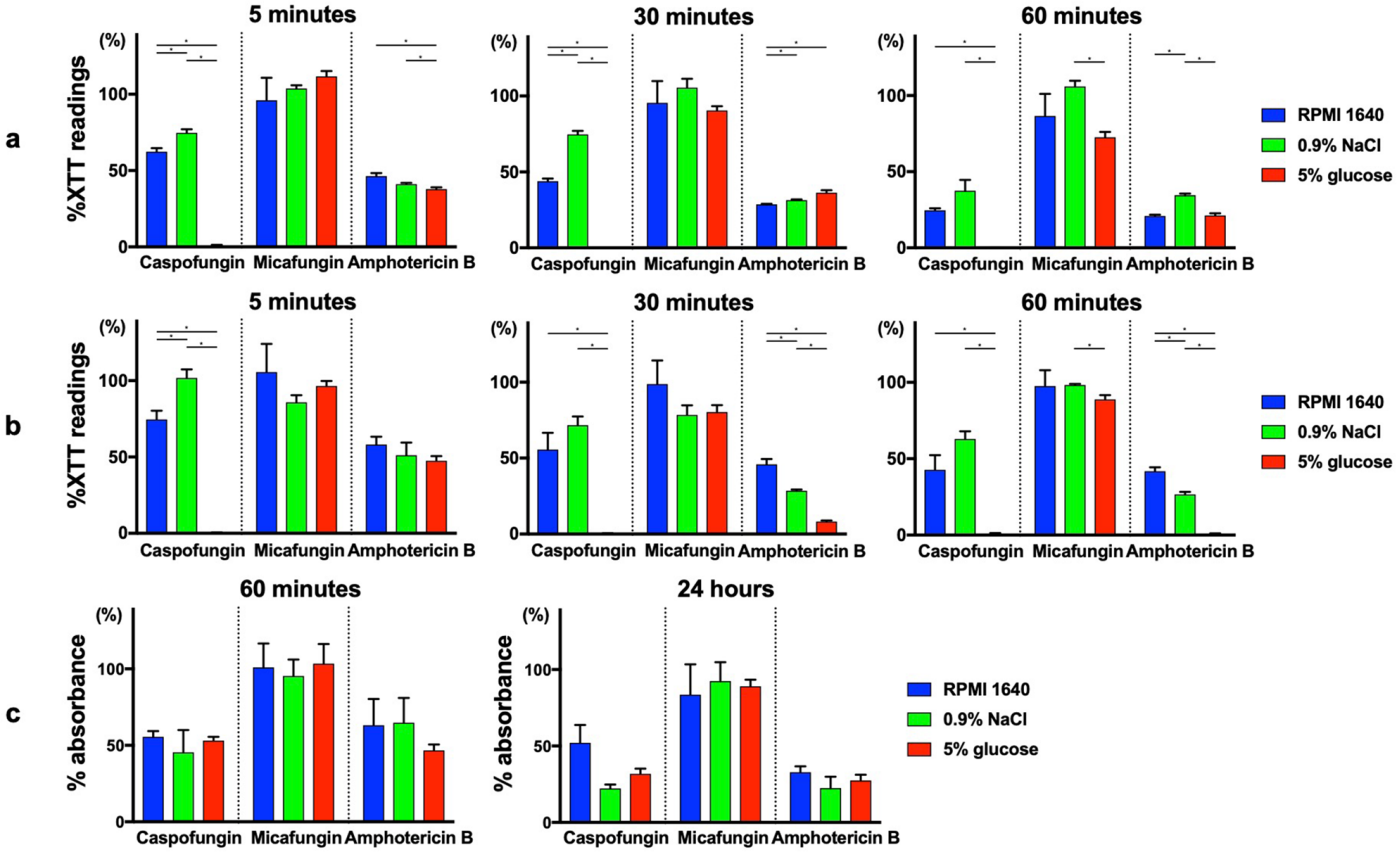
XTT assay and crystal violet assay of *Candida* biofilm cells treated with antifungals in different media and solutions. XTT assay of (**a**) *C. albicans* and (**b**) *C. auris* biofilm cells after 5 min, 30 min, and 60 min of antifungal treatment, respectively. Crystal violet assay of (**c**) *C. albicans* biofilm cells after 60 min and 24 h of antifungal treatment. Values are expressed as average percent readings (± SD) relative to control wells containing antifungal-free solution. *p < 0.0056, Bonferroni adjustment.

**Figure 3:**
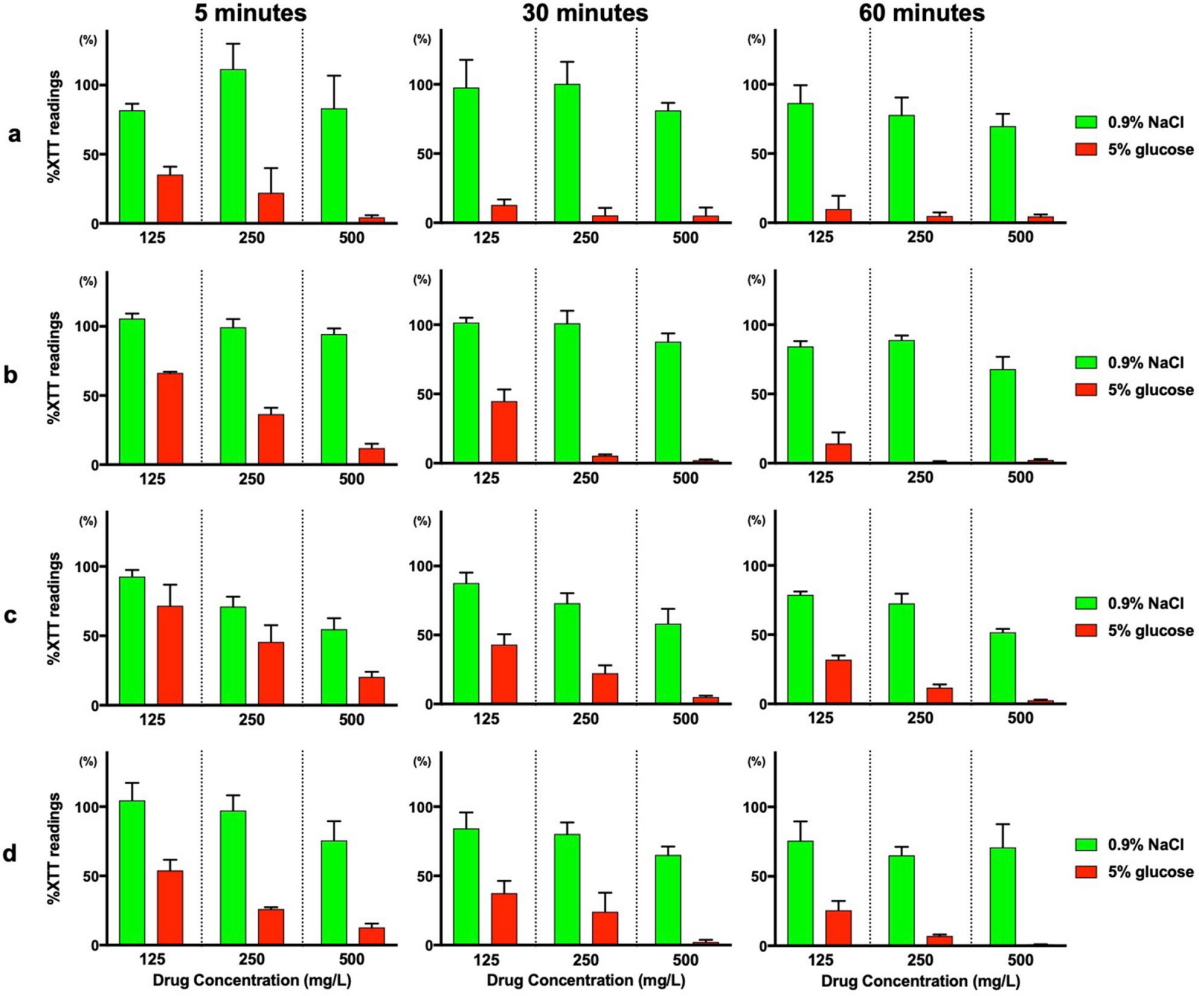
XTT reduction assay of bacterial and polymicrobial biofilm cells treated with caspofungin. XTT reduction assay of (**a**) MSSA, (**b**) MRSA, (**c**) *C. albicans* + MSSA, and (**d**) *C. albicans* + MRSA biofilm cells after treatment with caspofungin dissolved in 0.9% NaCl and 5% glucose water at the indicated concentrations for 5 min, 30 min, or 60 min, respectively. Values are expressed as average percent readings (± SD) relative to control wells containing antifungal-free solution.

We evaluated the effect of antifungal drugs dissolved in different solutions on *Candida* and bacterial biofilm cells. *C. albicans* and *C. auris* biofilm cells were exposed to caspofungin, micafungin and amphotericin B at a concentration of 500 mg/L for 5, 30, and 60 min. Treatment with caspofungin dissolved in 5% glucose significantly reduced both *C. albicans* (Fig. 2a) and *C. auris* (Fig. 2b) biofilm cells in 5 min with > 99% 2,3-bis(2-methoxy-4-nitro-5-sulfo-phenyl)-2*H*-tetrazolium-5-carboxanilide (XTT) reduction*,* compared to caspofungin dissolved in Roswell Park Memorial Institute (RPMI)-1640 or 0.9% NaCl which did not exert such rapid and potent antibiofilm effect (p < 0.0056, Bonferroni adjustment). This antibiofilm effect of caspofungin dissolved in 5% glucose was not observed in micafungin and was greater than seen in amphotericin B. Various antifungal concentrations were tested, and similar results were observed where caspofungin dissolved in 5% glucose had greater antibiofilm effect than other solutions (Supplementary Figs. S3 and S4). Additionally, crystal violet staining assay was performed to assess the effect of caspofungin on the biofilm mass, in contrast to the XTT assay which measures the metabolic activity of cells. After 60 min or 24 h of antifungal treatment, no significant difference was observed among RPMI-1640, 0.9% NaCl, and 5% glucose water in crystal violet staining (Fig. 2c), or at various antifungal concentrations (Supplementary Fig. S5). These results indicated that caspofungin dissolved in 5% glucose water had no activity against biofilm layers, but rather had direct effects on the cells inside the biofilm. This antibiofilm activity was further observed on methicillin-sensitive *Staphylococcus aureus* (MSSA), MRSA, and polymicrobial biofilm cells (*C. albicans* + MSSA, *C. albicans* + MRSA, *C. auris* + MSSA, *C. auris* + MRSA). There was > 80% XTT reduction in 5 min and > 95% XTT reduction in 60 min for both single and dual species biofilms when caspofungin was dissolved in 5% glucose water at the concentration of 500 mg/L (Fig. 3 and Fig. S6).

**Figure 4:**
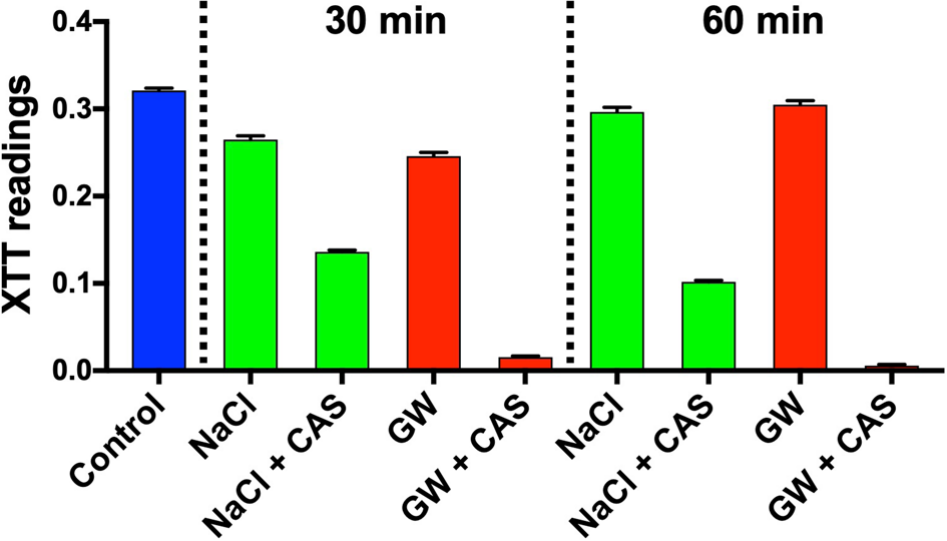
XTT reduction assay using clinically used central venous catheters. *C. auris* biofilm cells were grown in 14 G central venous catheter and treated with 0.9% NaCl (NaCl) and 5% glucose water (GW), with and without 125 mg/L caspofungin (CAS) for 30 min and 60 min, respectively. The catheters were then injected with XTT solution for spectrophotometric analysis at 492 nm.

The in vitro catheter-lock model was used for further evaluation on biofilm cells. When caspofungin was dissolved in 5% glucose water at the concentration of 125 mg/L, there was 94% and 99% XTT reduction against *C. auris* biofilms cells at 30 and 60 min, respectively, compared to 49% and 66% XTT reduction when dissolved in 0.9% NaCl at 30 and 60 min, respectively (Fig. 4).

### Alteration of chemical shifts in caspofungin-specific nuclear magnetic resonance spectroscopy (NMR) spectra in the presence of NaCl, KCl, and Na_2_SO_4_ and initial geometry optimization

**Figure 5:**
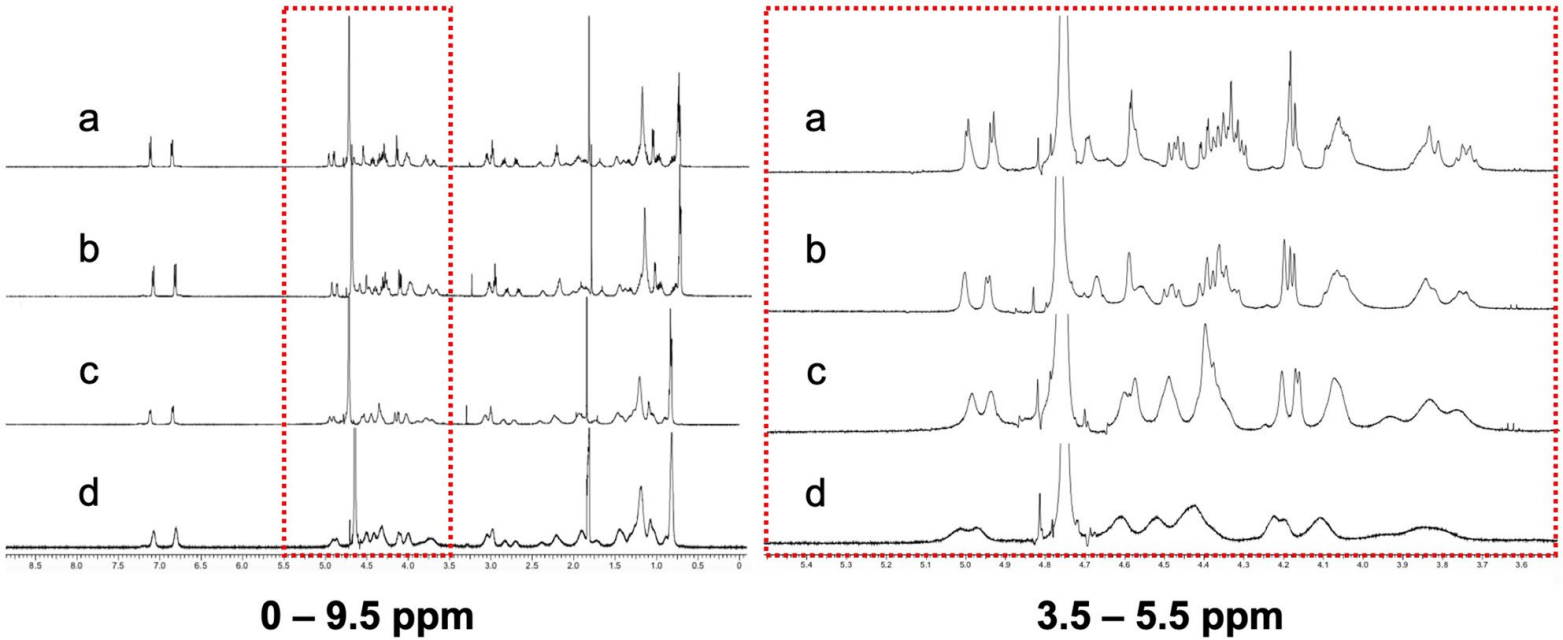
^1^H NMR spectra (500 MHz) of caspofungin in dH_2_O with and without NaCl. Caspofungin was dissolved in (**a**) dH_2_O, (**b**) NaCl (28 mM), (**c**) NaCl (125 mM), or (**d**) NaCl (417 mM). The left panel shows the scale at 0–9.5 ppm, and the right panel shows the red dotted squared section scaled to 3.5–5.5 ppm.

To explore interactions between echinocandins and sodium salts, we investigated chemical shifts of echinocandins in the presence and absence of NaCl via NMR analysis (Fig. 5). The presence of NaCl clearly changed the chemical shifts of caspofungin in a dose-dependent manner. The peak shapes for caspofungin became broader as NaCl concentration increased. In particular, we observed large changes in peaks ranging from 4.0 ppm to 4.7 ppm and assigned them to protons adjacent to the –NH–CO– moiety of the cyclic peptide backbone and hydroxyl groups. Thus, it is most likely that ions interact with the carbonyl, amino, or hydroxyl groups of caspofungin through coordinate bonding that causes conformational changes in the molecule. The changes observed in the caspofungin NMR spectra were not evident for micafungin or anidulafungin (Supplementary Fig. S7). Further NMR analyses of caspofungin plus KCl or Na_2_SO_4_ showed similar chemical shifts as those observed for NaCl (Supplementary Fig. S8). To study the stable conformation of caspofungin, we performed density functional theory calculations (FMO-HF/6-31G). These computational calculations showed that the initial geometry of caspofungin was formed by the internal hydrogen bond among three hydroxyl groups (Supplementary Fig. S9a–c). Taken together, our results show that the geometry of caspofungin under low ionic conditions may have an impact on intracellular penetration and antifungal activity. From NMR experiments, we observed that the geometry was changed by ions in a concentration dependent manner.

### Increased intracellular accumulation of fluorescein isothiocyanate (FITC)-caspofungin, intracellular reactive oxygen species (ROS) level, and cell death in low ionic conditions

**Figure 6:**
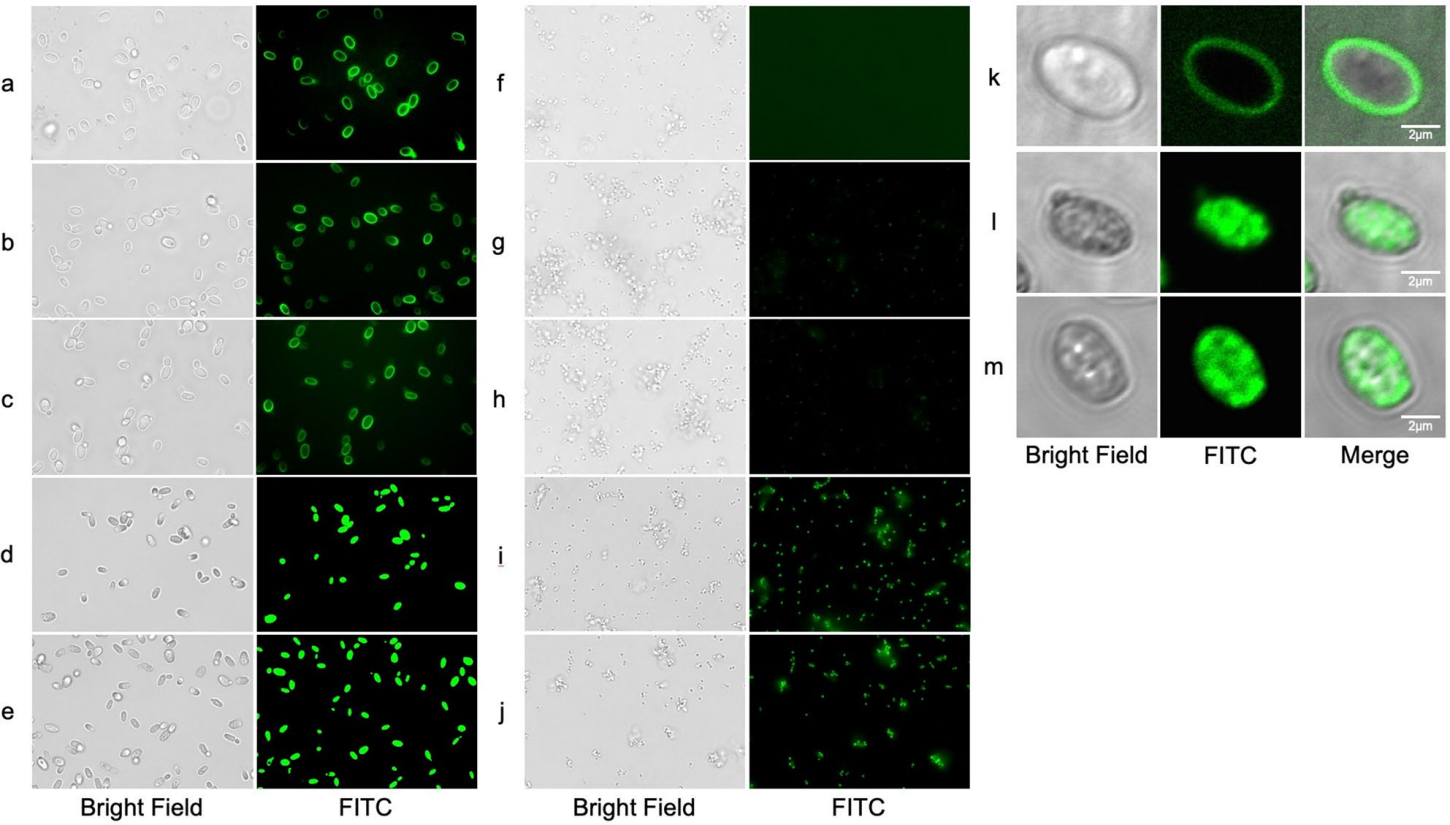
Fluorescent imaging of FITC-caspofungin accumulation patterns in *C. glabrata* and MRSA. Observed with a digital microscope, *C. glabrata* was exposed to FITC-caspofungin dissolved in (**a**) RPMI-1640, (**b**) 0.9% NaCl, (**c**) 0.9% KCl, (**d**) dH_2_O, and (**e**) 5% glucose water, MRSA was exposed to FITC-caspofungin dissolved in (**f**) RPMI-1640, (**g**) 0.9% NaCl, (**h**) 0.9% KCl, (**i**) dH_2_O, and (**j**) 5% glucose water. Observed with a confocal laser microscope, *C. glabrata* was exposed to FITC-caspofungin dissolved in (**k**) RPMI-1640, (**l**) dH_2_O, and (**m**) 5% glucose water.

To study the drug distribution in cells, *C. glabrata* and MRSA cells were exposed to FITC-caspofungin for 30 min and observed under a digital fluorescent microscope. In *C. glabrata*, accumulation at the peripheral borders of the cell was observed when FITC-caspofungin was dissolved in RPMI-1640, 0.9% NaCl, and 0.9% KCl (Fig. 6a–c), whereas a strong intracellular accumulation was observed when FITC-caspofungin was dissolved in dH_2_O and 5% glucose water (Fig. 6d,e). In MRSA, no accumulation was observed in RPMI-1640, 0.9% NaCl, or 0.9% KCl (Fig. 6f–h), whereas a strong intracellular accumulation was observed in dH_2_O and 5% glucose water (Fig. 6i,j). Additional detailed observations were made using a confocal laser scanning microscope. Clear peripheral accumulation in the cell borders was observed in RMPI-1640 (Fig. 6k). It is most likely that FITC-caspofungin accumulated at the cell membrane, since the target of caspofungin is β-1,3-d-glucan synthase, an integral protein of the cell membrane. In contrast, diffuse intracellular accumulation of FITC-caspofungin was observed without any specific localizations in dH_2_O and 5% glucose water (Fig. 6l,m).

**Figure 7:**
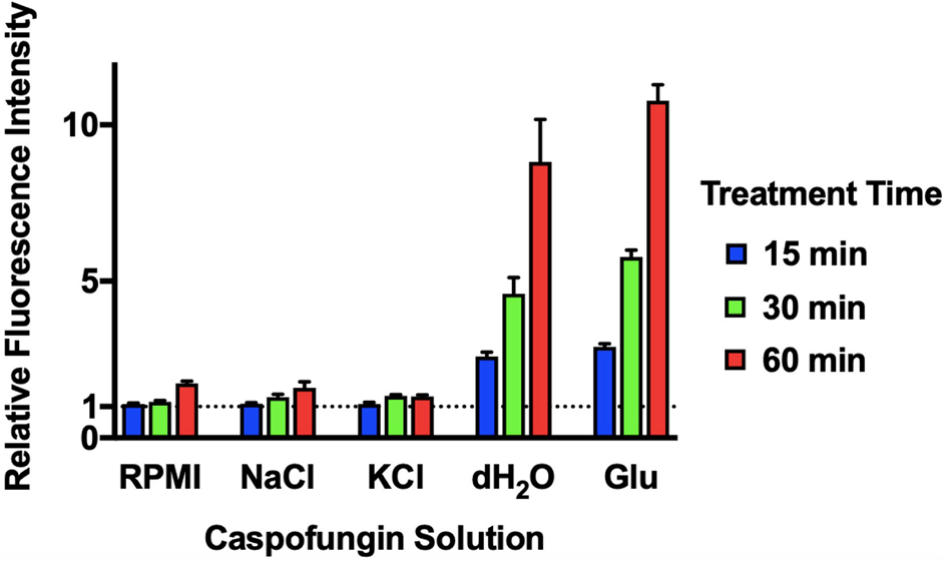
Intracellular ROS level of *C. glabrata*. *C. glabrata* cells that were treated with 50 μM H_2_DCFDA and exposed to 50 mg/L caspofungin dissolved in RMPI-1640, 0.9% NaCl, 0.9% KCl, dH2O, and 5% glucose water, respectively. Caspofungin-free solutions were used as controls. The intracellular ROS level was determined by measuring H_2_DCFDA conversion to fluorescent dichloroluorescein. Fluorescence intensity (FI) was measured at 15 min, 30 min, and 60 min. Relative FI (± SD) was calculated as caspofungin treated FI/control FI.

Next, the intracellular ROS levels in *C. glabrata* cells were measured by using 2′,7′-dichlorodihydrofluorescein diacetate (H_2_DCFDA) after treatment with caspofungin dissolved in various solutions (Fig. 7). Relative fluorescence intensity (RFI) showed double, quadruple, and octuple increase at 15 min, 30 min, and 60 min respectively, in caspofungin dissolved in dH_2_O and 5% glucose water, while RFI increase was limited with caspofungin dissolved in RPMI-1640, NaCl, and KCl solutions. Viability study at 30 min using methylene blue staining and colony forming units (CFU) counting showed that increases in intracellular FITC-caspofungin accumulation correlated with cell death (Supplementary Table S3). Percentages of intracellularly FITC-caspofungin accumulated cells for RPMI-1640, 0.9% NaCl, 0.9% KCl, dH_2_O, and 5% glucose water were 1.0%, 0%, 0%, 97.5%, and 99%, respectively, while methylene blue stain-positive cells for RPMI-1640, 0.9% NaCl, 0.9% KCl, dH_2_O, and 5% glucose water were 0.25%, 0%, 0%, 92.8%, and 96.3%, respectively. No significant change in CFU counts was observed in RPMI-1640, 0.9% NaCl, and 0.9% KCl, but CFU counts decreased by 91.9% and 99.6% in dH_2_O and 5% glucose water, respectively.

### Toxicity of caspofungin dissolved in 5% glucose water on human cells was similar to that of caspofungin dissolved in 0.9% NaCl and RPMI-1640

**Figure 8:**
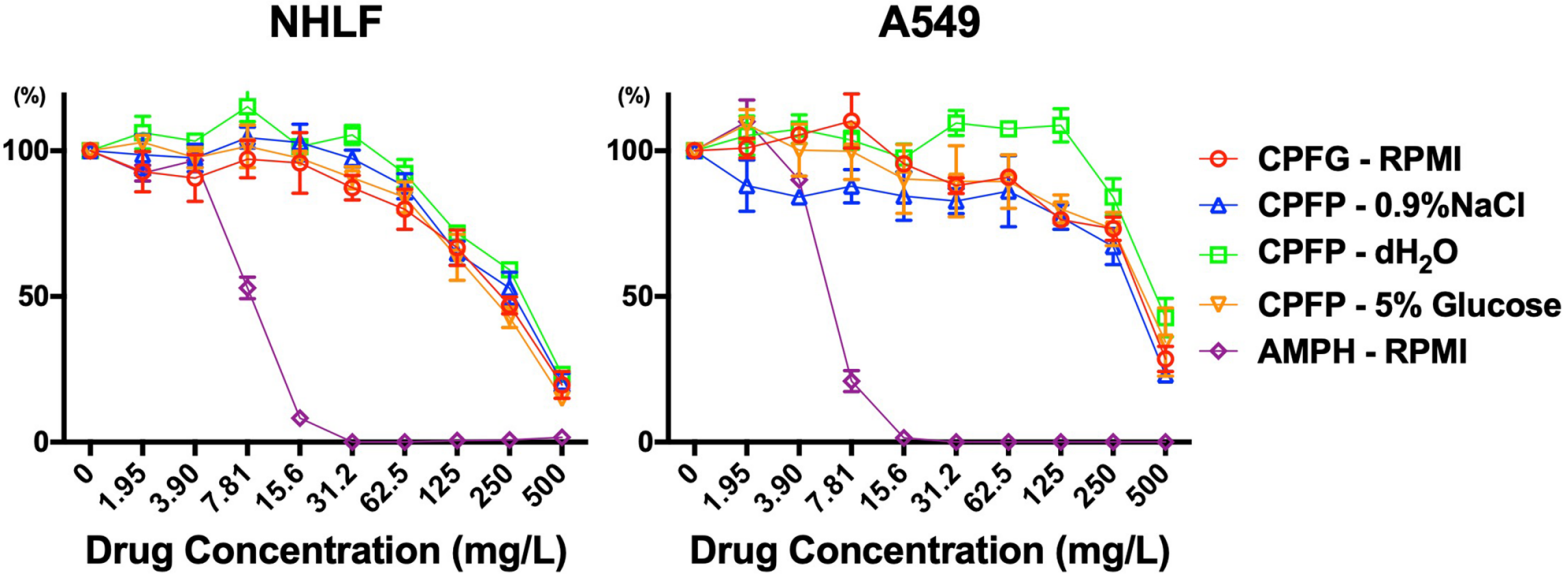
Toxicity evaluation of caspofungin solutions on human cell lines. NHLF and A549 cells were exposed to caspofungin dissolved in RPMI-1640, 0.9% NaCl, dH_2_O, and 5% glucose water, and amphotericin B dissolved in RPMI-1640 at various drug concentrations. Toxicity was evaluated by MTT cell viability assay.

Clonetics Human Lung Fibroblast (NHLF) and A549 cells were exposed to caspofungin dissolved in RPMI-1640, 0.9% NaCl, dH_2_O, and 5% glucose water, and amphotericin B dissolved in RPMI-1640 at various drug concentrations and evaluated by 3-(4,5-dimethylthiazol-2-yl)-2,5-diphenyl tetrazolium (MTT) cell viability assay (Fig. 8). Toxicity levels of caspofungin were similar in the four solutions where the half maximal effective concentrations (EC_50_) of caspofungin dissolved in RPMI, 5% glucose water, 0.9% NaCl, and dH_2_O were 497 mg/L, 310 mg/L, 294 mg/L, and 413 mg/L, respectively, and less than amphotericin B, which had an EC_50_ of 8.16 mg/L in NHLF cells. Similar results were observed in A549 cells. Amphotericin B at 31.2 mg/L contained 0.312% dimethyl sulfoxide (DMSO), which did not show cell toxicity at the same concentration or lower in an independent study (Supplementary Fig. S10).

## Discussion

Morbidity and mortality of biofilm infections including catheter-related candidemia and bacteremia remain unacceptably high with current antimicrobial agents^2,15–17^. In addition, multidrug-resistance and polymicrobial infection often make treatment difficult^8,9,18,19^. Removal of intravenous catheters is recommended but not always feasible^20,21^. As there are substantial hurdles to drug development, effective utilization of existing drugs is a reasonable option^22,23^. This study revealed a novel and unique antimicrobial capability of caspofungin that has the potential to overcome these difficulties. To the best of our knowledge, this is the first report describing the antimicrobial capacity of caspofungin against both MDR *Candida* and bacterial species.

Jiafeng et al.^24^, later followed by Chen et al.^25^ reported a similar phenomenon where aminoglycoside exhibited rapid and potent bactericidal effects under hypoionic conditions. However, they concluded that this effect was due to the enhancement of the conventional mechanism of action (binding of aminoglycoside to its conventional target 30S ribosome enhanced through ion channel activation under hypoionic conditions), and this effect was less potent against aminoglycoside resistant strains. We hypothesize that the effect of caspofungin dissolved in low ionic solutions involves a mechanism of action different from the inhibition of β-1,3-d-glucan biosynthesis, based on the extremely rapid fungicidal activity under static conditions and the broad antimicrobial effect against echinocandin-resistant species including bacteria. FITC-caspofungin also showed distinct patterns of drug accumulation in low ionic solutions (diffuse intracellular accumulation) compared to that in RPMI-1640 (cell border accumulation). Increased intracellular accumulation and rapid cell death under hypoionic conditions were observed in both *Candida* and bacterial cells in a similar manner, suggesting a universal activity against both organisms. Furthermore, this is an off-target effect of caspofungin, because the conventional drug target β-1,3-d-glucan synthase is absent in bacteria. However, the exact mode of action, such as intracellular target molecule of caspofungin and mechanisms of cell death under this specific condition, remains to be elucidated.

Our NMR study showed that caspofungin changes its molecular structure in an ion concentration-dependent manner, which may have altered the cell penetration property of the drug. Thus, a most likely reason for why only caspofungin among echinocandins shows antimicrobial action is that only caspofungin changes its chemical form under low ionic conditions. Rezai et al. reported that the entropy of cyclic peptides affects membrane diffusion and affinity for target proteins via internal hydrogen bonds^26^. The increased intracellular penetration/accumulation of caspofungin under low ionic conditions was strongly associated with high ROS production and rapid cell death. Satish et al. reported that caspofungin induces ROS production, while other echinocandins induce ROS weakly or not at all most likely due to poor cell penetration^27^. In addition, it is widely recognized that ROS production induced by antifungals or other chemicals contributes to the killing of *Candida* planktonic and biofilm cells^28–31^. We therefore propose that the intracellular accumulation of caspofungin caused high ROS production as an off-target effect, which may have induced extreme oxidative stress and ultimately led to rapid cell death. Further study on the molecular structure of caspofungin and understanding the chemical functions in association with its transformation may lead to new discoveries that would help promote antimicrobial drug development.

In respect to utilizing this effect for clinical implication, it is noteworthy that caspofungin in 5% glucose water displayed rapid and potent activity not only to planktonic cells but also to biofilm cells with broad antimicrobial spectrum. Simitsopoulou et al., using a similar XTT assay method, demonstrated that caspofungin at concentrations of 256–2048 mg/L has a high antibiofilm activity against *C. albicans*, *Candida lusitaniae*, and *Candida guilliermondii*^32^. However, total eradication was achieved at 24 h. A recent review of antifungal lock solutions suggested that biofilm eradication should be achieved in less than 12 h of lock duration in order to allow patients to continue other treatments or parenteral nutrition, but the authors concluded that past studies rarely achieved total eradication, and even when achieved, the lock durations were often 24 h or more^14^. Our studies have also revealed a potent antimicrobial effect on biofilm cells using actual clinical catheter surfaces, which enhances the possibility of promoting new approaches to treating catheter-related infections, especially in patients without access to immediate catheter removal.

There are several issues requiring discussion when considering 5% glucose-caspofungin as a catheter lock solution. First, the safety of using this solution in catheters is an obvious concern, considering its extraordinarily broad spectrum of antimicrobial activity. Also, according to the manufacturer, caspofungin is considered unstable in diluents containing dextrose and is not administered intravenously under such conditions, although the definition of "unstable" remains obscure and data supporting this matter have not been revealed by the manufacturer^33^. This 5% glucose-caspofungin lock solution, which is not intended for infusion, is similar to the ethanol lock solution in some aspects as ethanol has been shown to be effective against biofilm pathogens in vitro^34,35^. It is also contraindicated for intravenous administration and past clinical studies have reported numerous cases of local and systemic alcohol toxicity from ethanol leakage^36,37^. In our toxicity assay using human cells, 5% glucose-caspofungin had no greater toxicity than caspofungin dissolved in 0.9% NaCl after the solution had been exposed to cell culture solution (10%FCS/RPMI). Combined with our time-kill assay results, we conclude that after caspofungin in hypoionic solution has been exposed to ionic compounds, it loses its broad antimicrobial spectrum and will exhibit the same toxicity as caspofungin dissolved in ionic solutions such as 0.9% NaCl or RPMI-1640. We were not able to evaluate the toxicity of caspofungin in pure 5% glucose water or dH_2_O because we could not produce such an environment for human cells (mammalian cells, unlike fungal cells, were not able to survive in such conditions). However, this evaluation may not be necessary since human cells do not exist in such conditions in the body. These results support our theory that a leak of a small dosage from the catheter into the blood stream will not have direct toxic effects on the endothelial cells that line the blood vessels after it has been exposed to the blood serum containing rich ions. Caspofungin is also less likely to induce systemic toxicities than ethanol, considering it is a widely used antifungal with a relatively low rate of adverse effects. Therefore, utilizing this unique feature of caspofungin may reduce some difficulties that are currently being experienced with ethanol lock solutions. However, in vivo experiments are required to make more precise assessments on the efficacy and toxicity of 5% glucose-caspofungin solution.

In conclusion, this study demonstrated that caspofungin exhibits potent antifungal and antibacterial effects under low ionic conditions. With its ability to reduce MDR *Candida* and *S. aureus* biofilm cells in a remarkably short time, future studies elucidating the precise mechanisms associated with this activity along with in vivo studies may promote the development of a new strategy to catheter-related infections.

## Methods

### Fungal and Bacterial strains, human cell lines, and culture conditions

The following microbial strains were used in this study: wild-type *Candida albicans* ATCC MYA-2876/SC5314, *C. glabrata* ATCC2001/CBS138, *C. auris* NCPF8984 and NCPF8985^38^, MSSA ATCC25923, and MRSA ATCC43300. *C. glabrata* CGL305 (source: blood), MRSA40 (source: bronchoalveolar lavage fluid), and MDRP1481 (source: urine) were clinical isolates at the Nagasaki University Hospital (Nagasaki, Japan). The following human cell lines were used in the toxicity study: human lung carcinoma A549 cells (ATCC CCL-185) and NHLF cells (Lonza Walkersville, Inc., MD, USA). *Candida* and bacterial strains were maintained in YPD broth and Tris-buffered saline containing 25% glycerol, respectively, and frozen at − 70 °C until use. Human lung carcinoma A549 cells and NHLF cells were stored in RPMI-1640 containing 10% fetal calf serum and Fibroblast Basal Medium containing 10% fetal calf serum, respectively, and frozen with liquid nitrogen until use.

### Antimicrobials, reagents, and media

Caspofungin from two different sources was used to verify the consistency of our reported phenomena: caspofungin diacetate from Sigma-Aldrich (St. Louis, MO, USA) and caspofungin acetate from Merck (Rashway, NJ, USA). Micafungin sodium was provided by Astellas Pharma Inc. (Chuo-ku, Tokyo, Japan). Anidulafungin and amphotericin B were purchased from Sigma-Aldrich. Caspofungin and micafungin was dissolved in distilled water, amphotericin B and anidulafungin was dissolved in 100% DMSO. Glucose [d-(+)-glucose] was purchased from FUJIFILM Wako Pure Chemical Corporation (Osaka, Japan). Three types of dH_2_O were used in all experiments in order to verify the consistency of our reported phenomena: Invitrogen UltraPure DNase/RNase-free dH_2_O, Otsuka dH_2_O for 100% injection, and tap water purified using a Yamato Scientific Auto Still water purifier (WG250 with a 0.22 μm Sartolab RF filter; Sartorius, Göttingen, Germany).

### Antifungal susceptibility of planktonic cells and biofilm cells

The antifungal susceptibility of planktonic-form *Candida* (MICs) was determined using the Sensititre YeastOne colorimetric susceptibility test according to the manufacturer's instructions. MICs were defined as the lowest concentration of an antifungal agent that inhibits growth. SMICs (SMIC_50_ and SMIC_90_) of antifungals for *C. albicans* and *C. auris* biofilm cells were determined by using a 96-well flat-bottomed microplate (Iwaki microplate, 96 wells; Asahi Glass CO., LTD, Tokyo, Japan) as previously described with several modifications^39^. Briefly, 100 µL of *Candida* cells (1–5 × 10^6^ CFU/mL) in RPMI-1640 solutions were inoculated into each well of a 96-well microplate and incubated at 37 °C for 24 h for biofilm formation. Wells were washed and filled with 100 μL RPMI-1640 containing antifungal solutions at different concentrations and incubated at 37 °C for 24 h. The wells were then filled with 100 μL XTT solution (500 mg/L XTT sodium salt in sterile PBS) containing 1:10,000 (v/v) menadione solution (10 mM menadione in 100% acetone). The plates were then incubated in the dark at 37 °C for 2 h followed by spectrophotometric analysis at 492 nm (Multiskan FC microplate photometer; Thermo Fisher Scientific, Waltham, MA, USA). The lowest concentrations associated with a ≥ 50% and > 90% reduction compared to the control well represented the SMIC_50_ and SMIC_90_, respectively^40^.

### Time-kill assay on planktonic cells

Time-kill assay was performed according to previous reports, with some modifications^41^. Briefly, *Candida* and bacterial cultures at logarithmic phase were diluted to 1‒5 × 10^5^ CFU/mL with different media and solutions. Drugs were added to each 10 mL solution and were spread on agar to allow CFU counting at each time point (0 min, 30 min, 4 h, 8 h, and 24 h). Fungicidal and bactericidal effect was defined as a kill of ≥ 10^3^ CFU/mL from the starting inoculum. Fungistatic and bacteriostatic activity was defined as a growth-inhibitory effect and a kill of < 10^3^ CFU/mL (3 log_10_ CFU/mL) from the starting inoculum. The lowest detectable colony count was 50 CFU. Triplicate samples were analyzed at all time-points, in all experiments, and each experiment was performed independently at least twice.

### XTT reduction assay and crystal violet staining assays on biofilm cells

XTT assay and crystal violet staining assay were performed in 96-well microplates as previously described with several modifications^39,42,43^. For biofilm production, 100 µL of *Candida* cells (1–5 × 10^6^ CFU/mL) and *S. aureus* cells (OD_600_ of 0.15 equivalent to approximately 5 × 10^7^ CFU/ml) in RPMI-1640, respectively, were inoculated into each well of a 96-well microplate and incubated at 37 °C for 24 h. Glucose was additionally applied to *S. aureus* cells (final glucose concentration of 2%) to achieve maximum biofilm production. For dual-species biofilm formation, 100 µL of *Candida* cells and 100 µL of MSSA or MRSA cell solution was inoculated into each well.

For antifungal treatment, 100 μL of antifungal solutions (caspofungin, micafungin, and amphotericin B dissolved in 5% glucose water, 0.9% NaCl, and RPMI-1640, respectively) were added to each well and incubated at 37 °C for 5 min, 30 min, or 60 min. A maximum concentration was selected for each drug according to the clinical dose indicated for fungal infections and corresponding to intravenously-administered intracatheter concentrations: 500 mg/L for caspofungin (50 mg/body weight diluted with 100 mL or 250 mL solution); 1000 mg/L for micafungin (100 mg/body weight diluted with 100 mL solution); 2000 mg/L for amphotericin B (6 mg/kg/body diluted with 250 mL solution for a recommended final concentration of 1000‒2000 mg/L). For the XTT assay, 100 μL of XTT solution was added to each well and incubated in the dark at 37 °C for 2–3 h followed by spectrophotometric analysis at 492 nm. For crystal violet staining, 100 μL of 0.1% (wt/v) crystal violet was added and incubated at room temperature for 10 min. Each well was washed twice with 300 μL dH_2_O and destained with 125 μL 100% ethanol at room temperature for 10 min followed by spectrophotometric analysis at 595 nm. Biofilm cell viability and biofilm quantification was calculated as %XTT readings and % absorbance of treated well relative to negative control well, respectively. Quadruplicate samples were analyzed for XTT experiments, and triplicate samples were analyzed for crystal violet staining. All experiments were independently performed at least twice.

XTT assays were additionally performed with an in vitro catheter-lock therapy model using clinically used central venous catheters (14 G × 30 cm, single lumen, SMAC SMAC Plus, Japan Covidien Corporation, Tokyo, Japan). For biofilm formation, *C. auris* cells in RPMI-1640 (OD_600_ of 0.15 equivalent to approximately 5 × 10^6^ CFU/ml) were injected into catheters previously coated with 10% FCS and incubated at 37 °C for 48 h. For treatment, catheters were filled with 0.9% NaCl and 5% glucose water, with and without 125 mg/L caspofungin for 30 min and 60 min, respectively. Control groups were catheters without any treatment. The catheters were then injected with XTT solution and incubated at 37 °C for 2 h. The *C. auris* cells in XTT solution were transferred to a 96-well plate and the absorbance was measured with a spectrophotometer at 492 nm. Experiments were independently performed three times.

### NMR spectra analysis and density functional theory calculations

Caspofungin, micafungin, and anidulafungin were dissolved in dH_2_O and NMR spectra were recorded under the presence of NaCl, KCl, and Na_2_SO_4_, respectively. Procedures and analyses were performed as previously described with several modifications^44,45^. Detailed information is given in the supplementary information.

### Preparation and fluorescence imaging of FITC-caspofungin and viability studies

FITC-caspofungin was prepared by the condensation of caspofungin (36 mg, 0.03 mM) and FITC (12 mg, 0.03 mM) in the presence of Hunig's base (9 μL, 0.05 mM) in N,N-dimethylformamide (Supplementary Fig. S11). Procedures were performed as previously described with several modifications^46^. Additional information on the preparation of FITC-caspofungin and the fluorescence imaging method is given in the supplementary information.

### Measurement of intracellular ROS levels

The intracellular ROS level in *C. glabrata* was determined by measuring H_2_DCFDA conversion to fluorescent dichloroluorescein (DCF) as described previously with minor modifications^47^. Additional information is given in the supplementary information.

### Cell toxicity assay

The toxicity of caspofungin to human cells was evaluated by preforming MTT assay on A549 and NHLF cells with some modifications^48^. NHLF and A549 cells were incubated at 37 °C in a 90% humidified atmosphere containing 5% CO_2_ and subcultured every 3–5 days. For experiments, 5 × 10^3^ cells/well (100 µL/well) were seeded onto 96-well flat-bottomed microplates and allowed to grow for 24 h. Caspofungin diluted with RPMI-1640, 0.9% NaCl, 5% glucose water, and dH_2_O, respectively, and amphotericin B (containing 5% DMSO at 500 mg/L) diluted with RPMI-1640 was applied to each well and incubated for 24 h. After incubation, wells were washed and filled with 150 µL of MTT solution (2000 mg/L MTT/PBS), and incubated for 2 h. Each well was filled with 100 µL of 100% DMSO and spectrophotometrically analyzed at 540 nm (Multiskan FC microplate photometer; Thermo Fisher Scientific). Cell viability was calculated as % absorbance of treated well relative to negative control well. Triplet samples were analyzed for each experiment, and all experiments were independently performed at least twice.

### Statistical analysis

Data was statistically analyzed in the XTT reduction assay and crystal violet staining assay on biofilm cells. Significant differences were assessed with Welch's *t* test, and significance levels were adjusted using Bonferroni’s method. Analyses were performed using JMP software (JMP Pro Version 14, SAS Institute Inc., Cary, NC, USA). EC_50_ calculations in the toxicity assay were performed using nonlinear regression dialog with GraphPad Prism (version 7) software (GraphPad Software, San Diego, CA, USA).

## Supplementary information

Supplementary file 1

## Data Availability

The data that support the findings of this study are available from the authors upon reasonable request.
