# Supplementary material for: Novel and potent antimicrobial effects of caspofungin on drug-resistant Candida and bacteria
Source: Sci Rep. 2020 Oct 20;10:17745. doi: 10.1038/s41598-020-74749-8 (PMC7576149; doi:10.1038/s41598-020-74749-8)
Supplement: Supplementary file 1 [file 41598_2020_74749_MOESM1_ESM.docx]

**Supplementary Information**

**-Supplementary Figures**

**-Supplementary Tables**

**-Supplementary Methods**

**Title:** Novel and potent antimicrobial effects of caspofungin on drug-resistant *Candida* and bacteria

**Authors:** Makoto Sumiyoshi,^1,2^ Taiga Miyazaki,^2,3,^* Juliann Nzembi Makau,^4^ Satoshi Mizuta,^5^ Yoshimasa Tanaka,^6^ Takeshi Ishikawa,^7^ Koichi Makimura,^8^ Tatsuro Hirayama,^2^ Takahiro Takazono,^2,3^ Tomomi Saijo,^2^ Hiroyuki Yamaguchi,^2^ Shintaro Shimamura,^2^ Kazuko Yamamoto,^2^ Yoshifumi Imamura,^2^ Noriho Sakamoto,^2^ Yasushi Obase,^2^ Koichi Izumikawa,^3^ Katsunori Yanagihara,^9^ Shigeru Kohno,^2^ and Hiroshi Mukae^1,2^

**Affiliations:**

^1^Department of Respiratory Medicine, Nagasaki University Graduate School of Biomedical Sciences, 1-12-4 Sakamoto, Nagasaki 852-8523, Japan

^2^Department of Respiratory Medicine, Nagasaki University Hospital, 1-7-1 Sakamoto, Nagasaki 852-8501, Japan

^3^Department of Infectious Diseases, Nagasaki University Graduate School of Biomedical Sciences, 1-7-1 Sakamoto, Nagasaki 852-8501, Japan

^4^Department of Molecular Microbiology and Immunology, Nagasaki University Graduate School of Biomedical Sciences, 1-12-4 Sakamoto, Nagasaki 852-8523, Japan

^5^Center for Bioinformatics and Molecular Medicine, Nagasaki University Graduate School of Biomedical Sciences, 1-12-4 Sakamoto, Nagasaki 852-8523, Japan

^6^Center for Medical Innovation, Nagasaki University, 1-7-1 Sakamoto, Nagasaki 852-8588, Japan

^7^Department of Chemistry, Biotechnology, and Chemical Engineering, Graduate School of Science and Engineering, Kagoshima University, 1-21-40 Korimoto, Kagoshima 890-0065, Japan

^8^Medical Mycology, Graduate School of Medicine, Teikyo University, 2-11-1 Kaga, Itabashi-ku, Tokyo 173-8605, Japan

^9^Department of Laboratory Medicine, Nagasaki University Hospital, 1-7-1 Sakamoto, Nagasaki 852-8501, Japan

**Supplementary Figures**

**
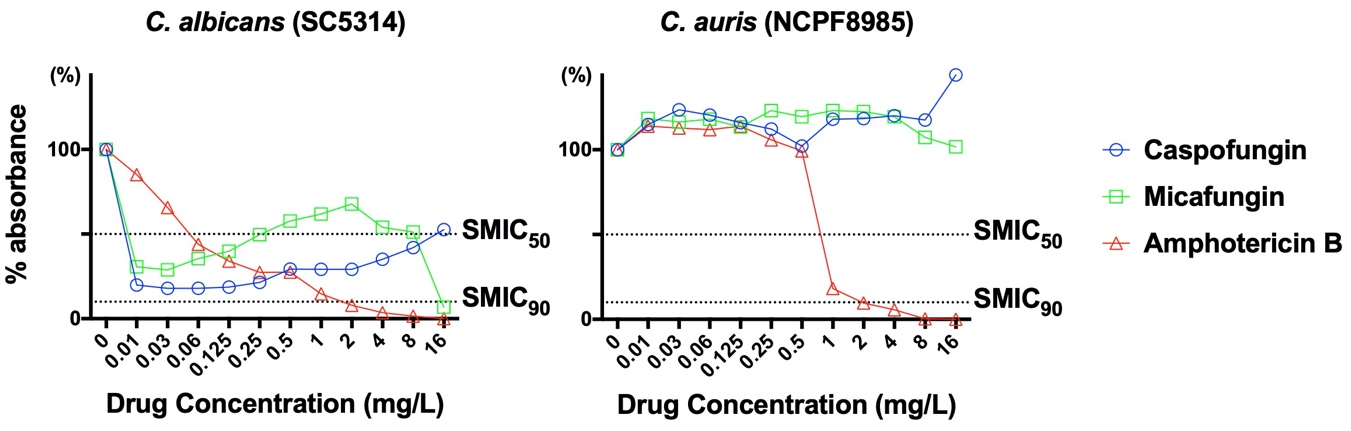
**

**Fig. S1** Activity of antifungals against pre-formed *C. albicans* and *C. auris* biofilms. *C. albicans* and *C. auris* were treated with antifungal solutions of different concentrations for 24 h. Paradoxical effects were observed following treatment with caspofungin and micafungin. Values are expressed as average percent readings relative to control wells containing antifungal-free solution.

**
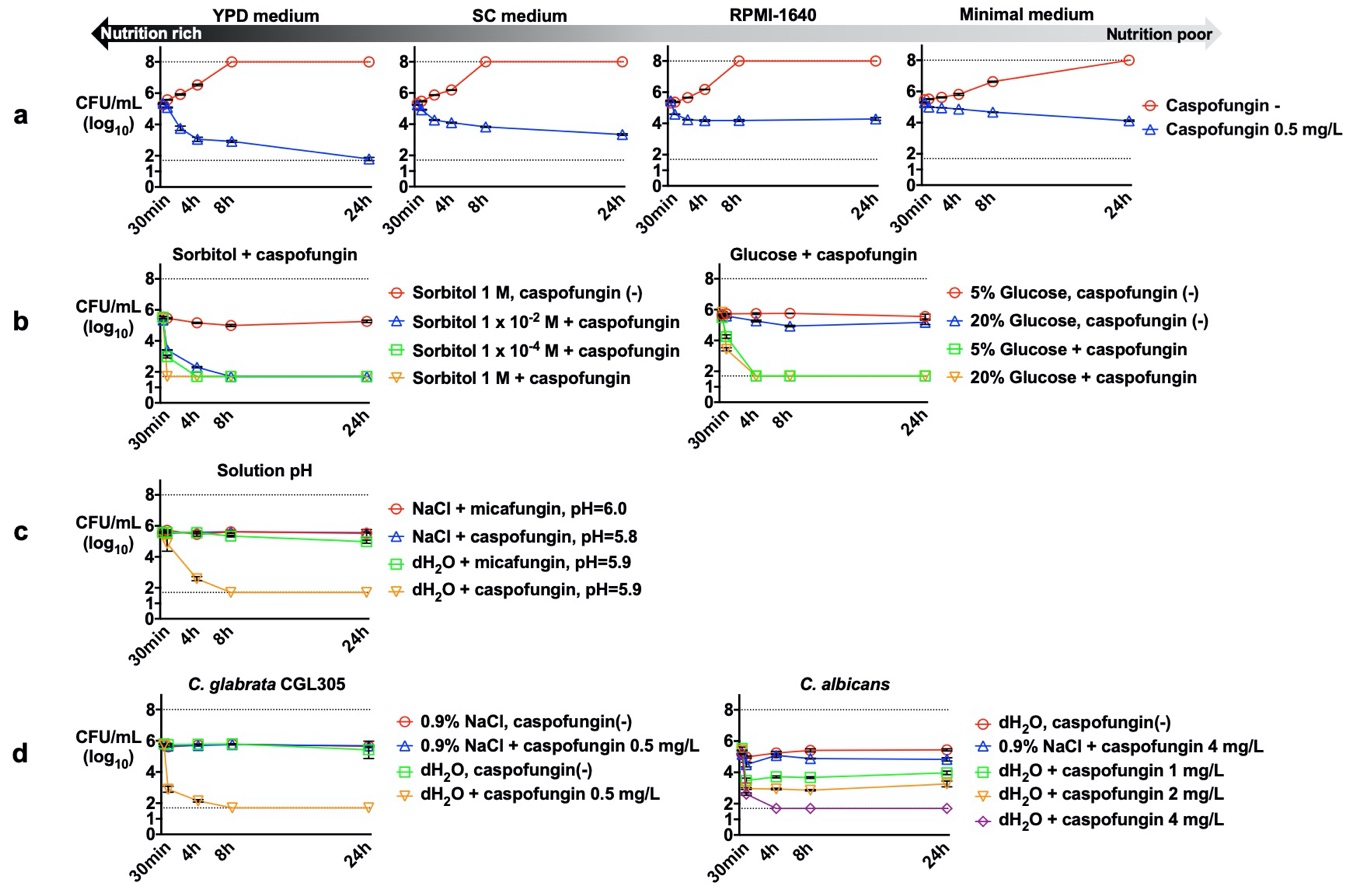
**

**Fig. S2** Time-kill assay of *Candida* cells using echinocandin drugs under various conditions. Antifungal effects of (**a**) caspofungin in growth media with different nutrient supply, (**b**) caspofungin under hyperosmotic conditions using sorbitol and glucose, (**c**) caspofungin, and micafungin at specific pH, and (**d**) caspofungin against *C. glabrata* CGL305 and *C. albicans*. Each data point represents the mean (± SD). Broken lines represent the limits of quantification at the upper (10^8^ CFU/mL) and lower limits (50 CFU/mL).

**
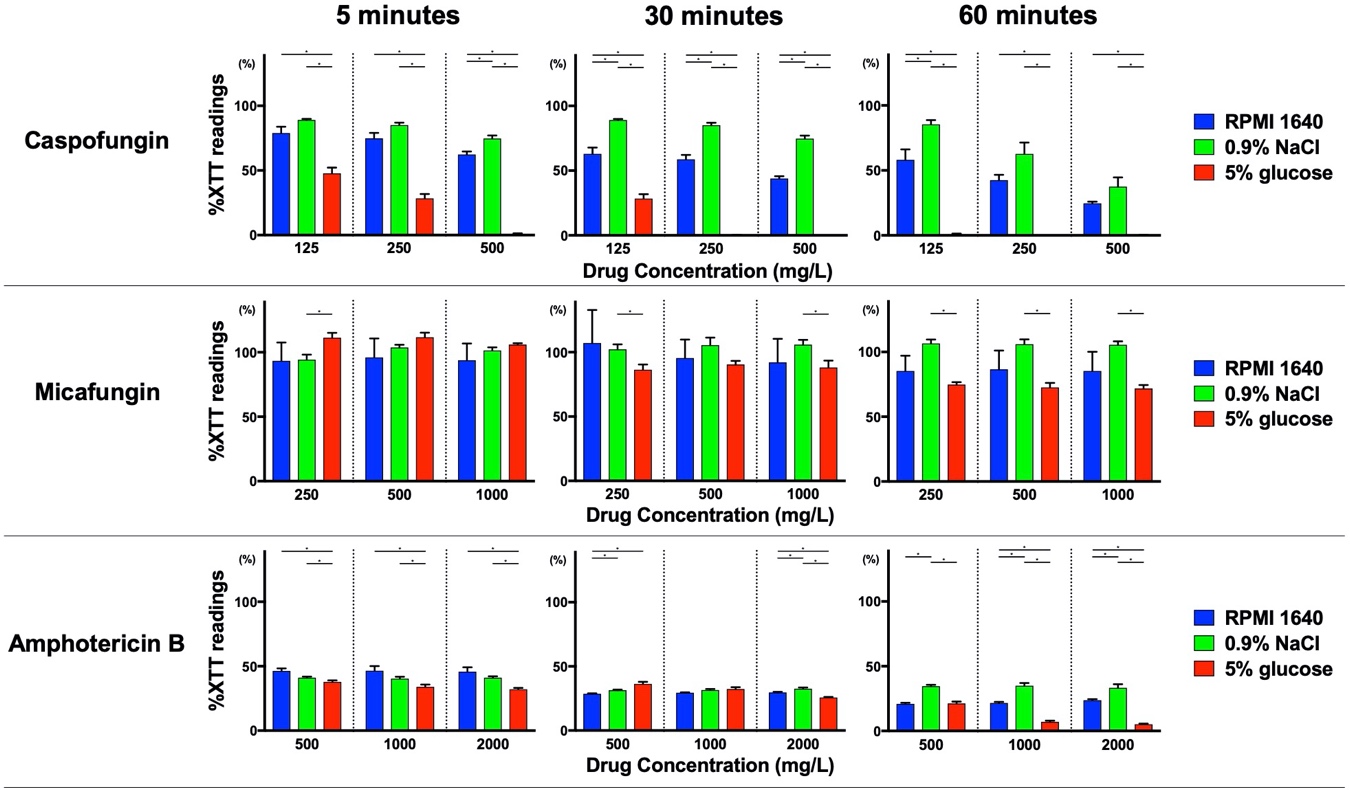
**

**Fig. S3** XTT assay of *C. albicans* biofilm cells treated with antifungals in different solutions and drug concentrations. *C. albicans* biofilm cells were treated with antifungal solutions at the indicated concentrations for 5 min, 30 min, and 60 min. Values are expressed as average percent readings (± SD) relative to control wells containing antifungal-free solution. *p < 0.0056, Bonferroni adjustment.

**
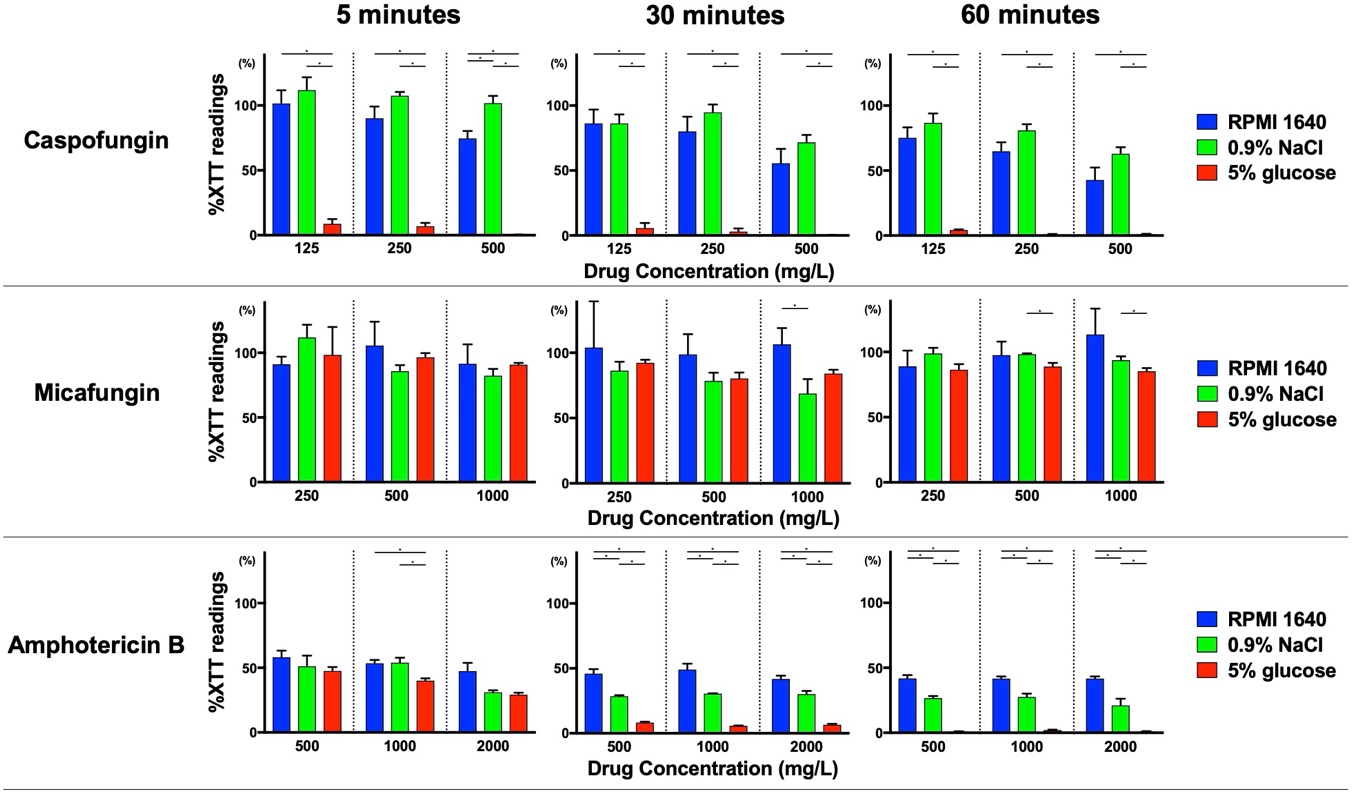
**

**Fig. S4** XTT assay of *C. auris* biofilm cells treated with antifungals in different solutions and drug concentrations. *C. auris* biofilm cells were treated with antifungal solutions at the indicated concentrations for 5 min, 30 min, and 60 min. Values are expressed as average percent readings (± SD) relative to control wells containing antifungal-free solution. *p < 0.0056, Bonferroni adjustment.

**
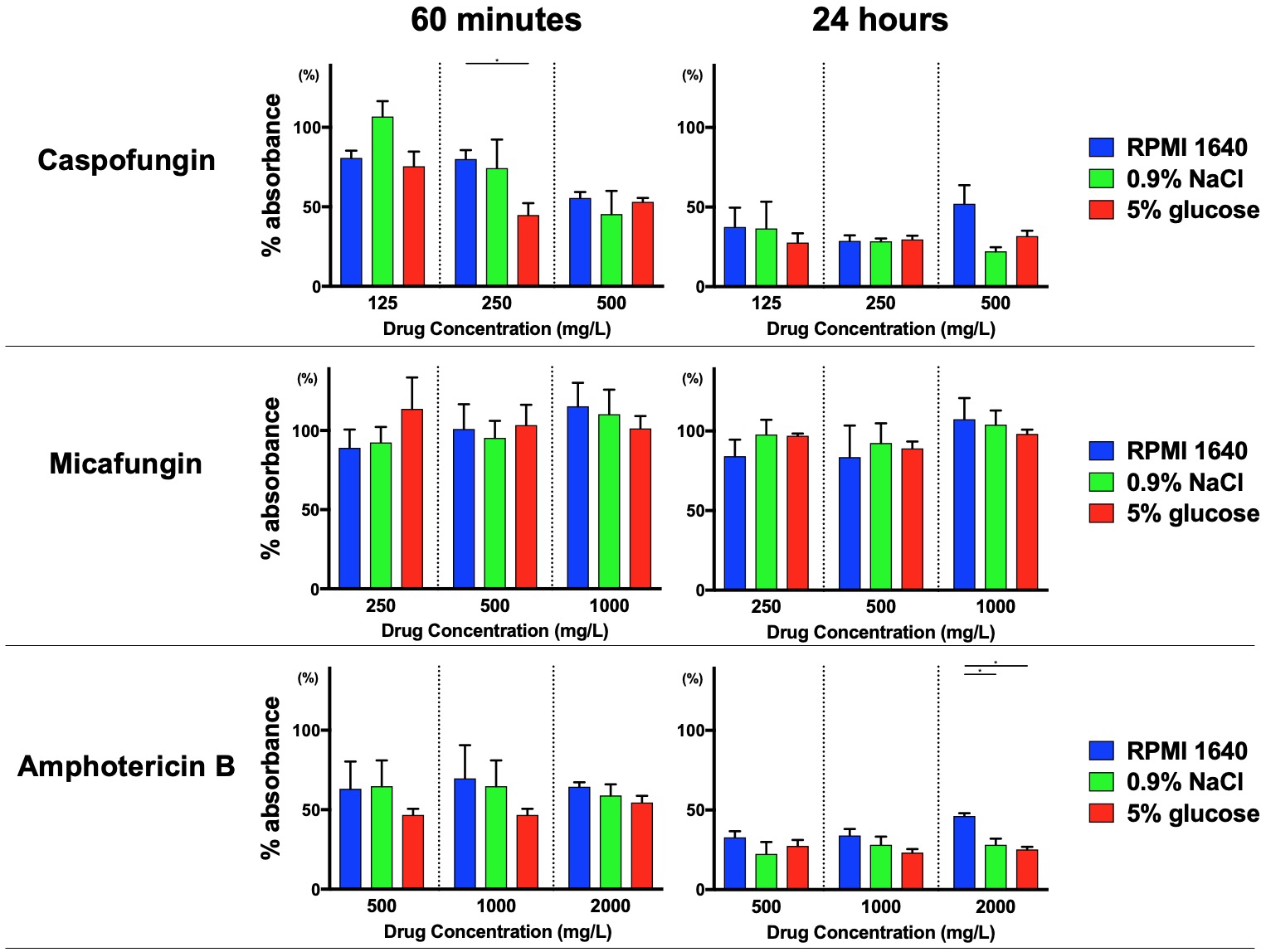
**

**Fig. S5** Crystal violet staining of *C. albicans* biofilm cells treated with antifungals in different media and solutions. *C. albicans* biofilm cells were treated with antifungal solution at the indicated concentrations for 60 min or 24 h. Values are expressed as average percent readings (± SD) relative to control wells containing antifungal-free solution. *p < 0.0056, Bonferroni adjustment.

**
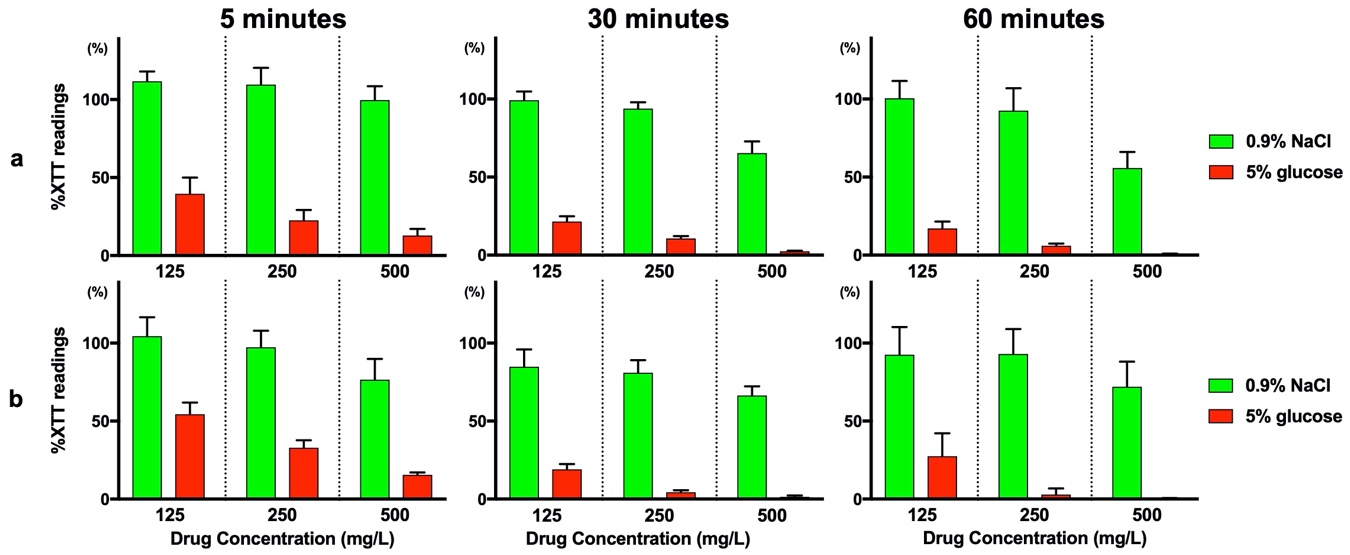
**

**Fig. S6** XTT reduction assay of polymicrobial biofilm cells treated with caspofungin. XTT reduction assay of (**a**) *C. auris +* MSSA and (**b**) *C. auris* + MRSA biofilm cells after treatment with caspofungin dissolved in 0.9% NaCl and 5% glucose water at the indicated concentrations for 5 min, 30 min, or 60 min, respectively. Values are expressed as average percent readings (± SD) relative to control wells containing antifungal-free solution.

**
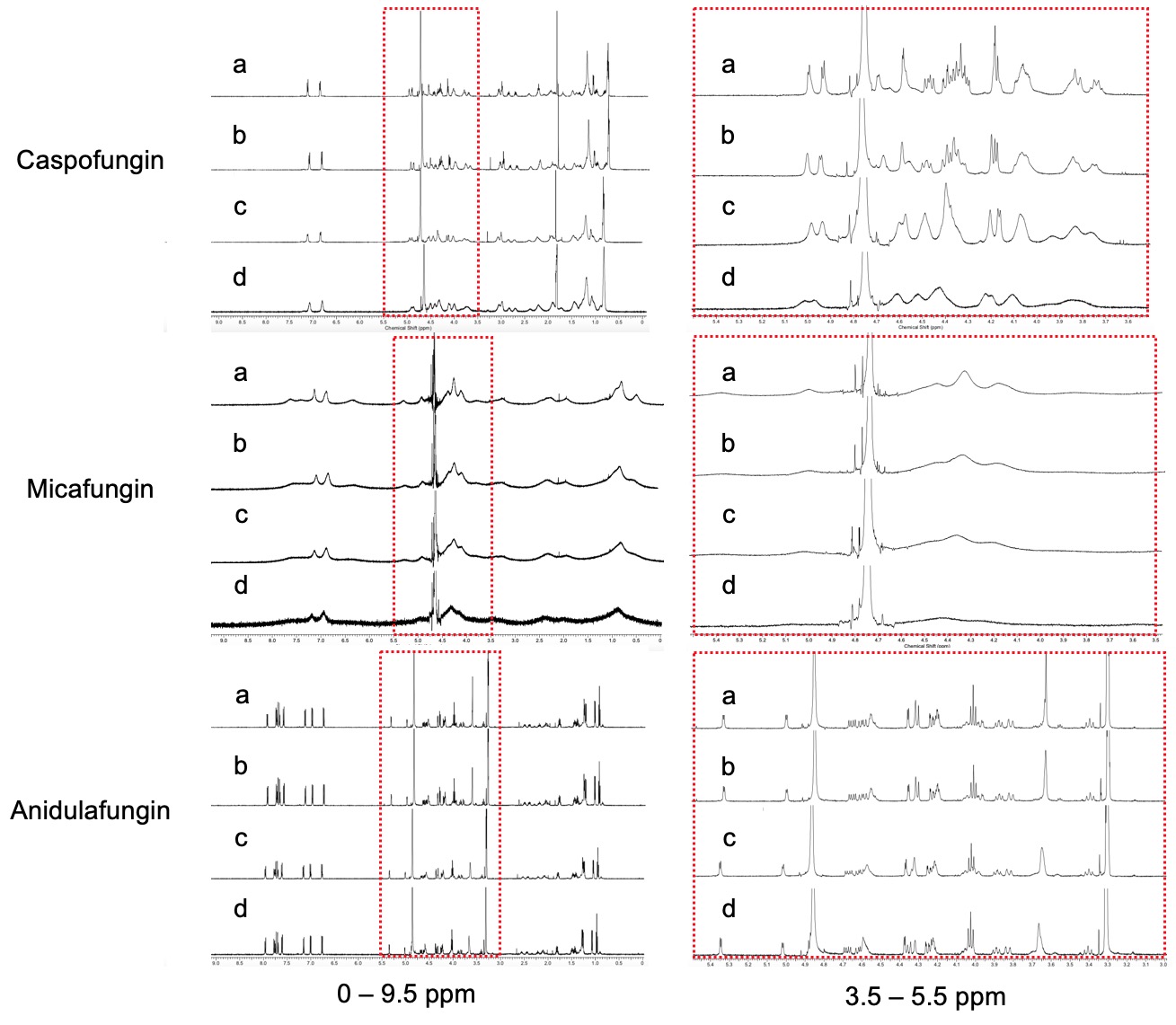
**

**Fig. S7** ^1^H NMR spectra (500 MHz) of caspofungin, micafungin, and anidulafungin in dH_2_O with and without NaCl. Caspofungin, micafungin, and anidulafungin were respectively dissolved in (**a**) dH_2_O, (**b**) NaCl (28 mM), (**c**) NaCl (125 mM), or (**d**) NaCl (417 mM). The left panel shows the scale at 0–9.5 ppm, and the right panel shows the red dotted squared section scaled to 3.5–5.5 ppm.

**
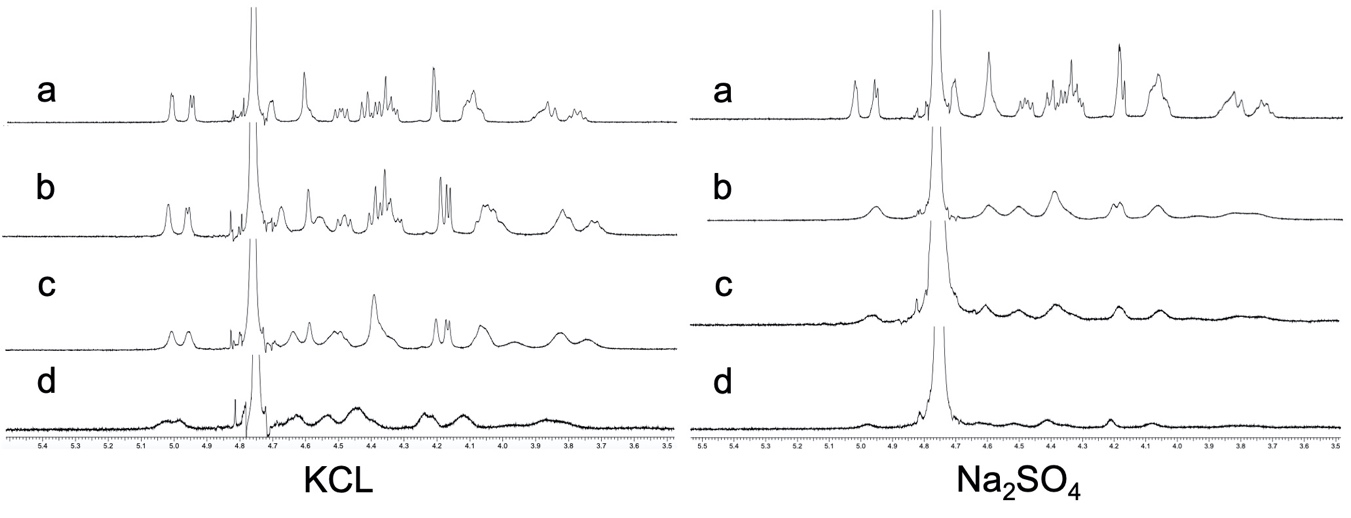
**

**Fig. S8** ^1^H NMR spectra (500 MHz) of caspofungin in dH_2_O with and without KCl or Na_2_SO_4_. Caspofungin was dissolved in (**a**) 0 mM, (**b**) 28 mM, (**c**) 125 mM, or (**d**) 417 mM KCl or Na_2_SO_4_.

**
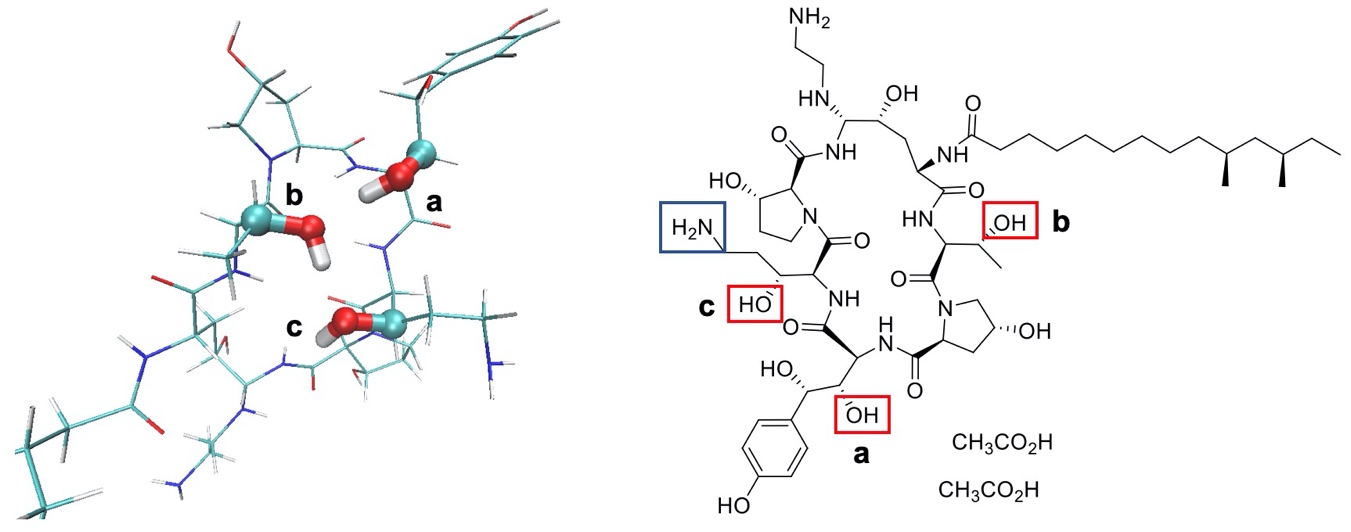
**

**Fig. S9** Optimized caspofungin structure. The optimal geometry of caspofungin is formed by internal hydrogen bonds among three hydroxyl groups (**a**, **b**, and **c**). The left panel shows the native conformation, and the right panel shows the chemical structure. Density functional theory calculations were performed using Molecular Operating Environment version 2014.09 software (MOLSIS, Inc., Montreal, Canada). Initial structures were generated and optimized with the Molecular Orbital PACkage (http://openmopac.net/). The geometries were further optimized with FMO-HF/6-31G.

**
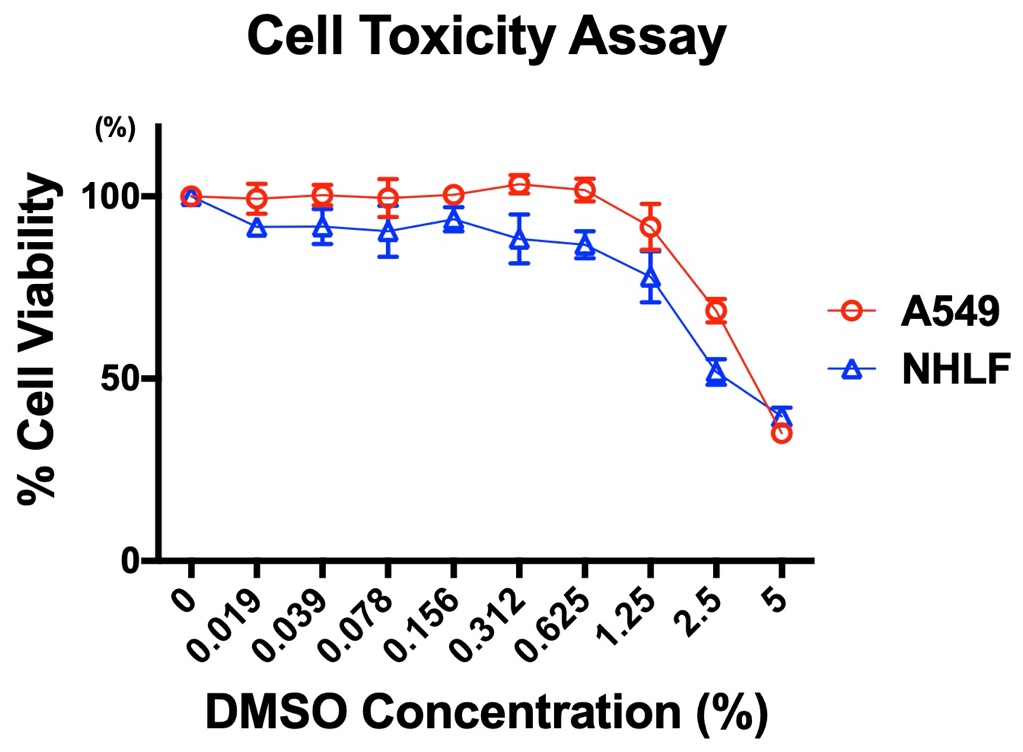
**

**Fig. S10** Cell toxicity of DMSO. DMSO was diluted with RPMI-1640 to a maximum concentration of 5% and its toxicity was evaluated on A549 and NHLF cells.

**
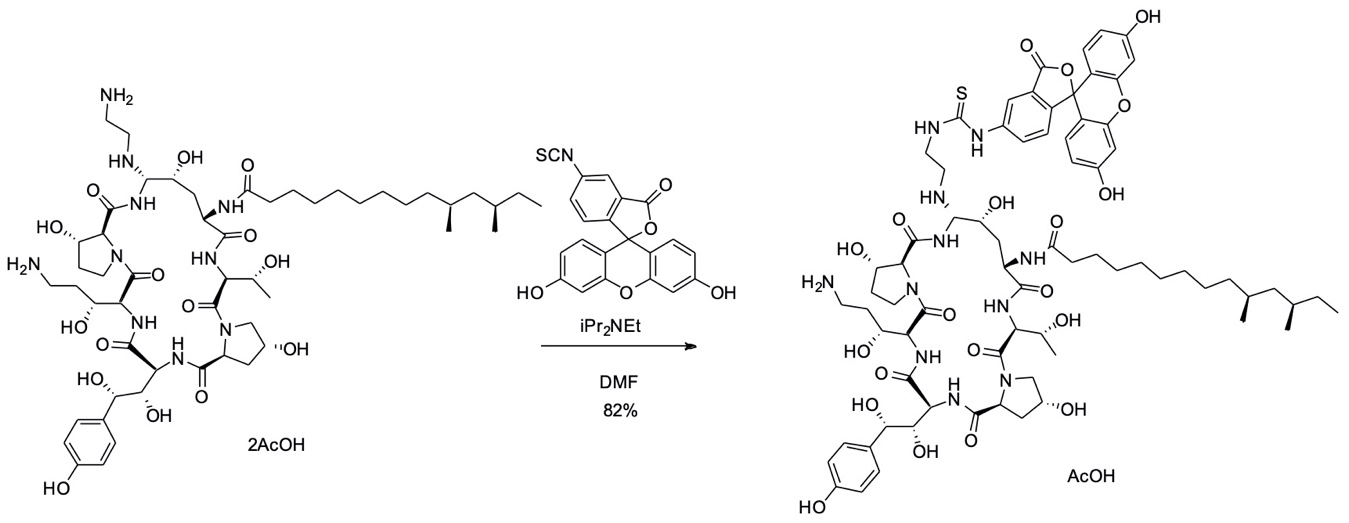
**

**Fig. S11** Conjugation of FITC-caspofungin. FITC-caspofungin was prepared by the condensation of caspofungin and fluorescein isothiocyanate in the presence of Hunig's base in DMF. Mass spectra were obtained on JEOL JMS-T100TD for electrospray ionization.

**Supplementary Tables**

**Table S1.** MICs of antifungals for *C. glabrata*, *C. albicans*, and *C. auris* planktonic cells

|  | MIC (mg/L) for planktonic cells^a^ | | | | |
| --- | --- | --- | --- | --- | --- |
|  | CPFG | MCFG | ANFG | FLCZ | AMPH-B |
| *C. glabrata* (CBS138) | 0.03 | 0.015 | 0.03 | 2 | 0.5 |
| *C. glabrata*  (CGL305) | 0.03 | 0.008 | 0.03 | 32 | 2 |
| *C. albicans* (SC5314) | 0.03 | 0.015 | < 0.015 | 0.25 | 0.5 |
| *C. auris* (NCPF8984) | > 8 | > 8 | > 8 | > 256 | 2 |
| *C. auris* (NCPF8985) | > 8 | > 8 | 4 | > 256 | 2 |

^a^MICs for planktonic cells were determined using the Sensititre YeastOne susceptibility test.

CPFG, caspofungin; MCFG, micafungin; ANFG, anidulafungin; FLCZ, fluconazole; AMPH-B, amphotericin B.

**Table S2.** MICs of antifungals for *C. albicans* and *C. auris* biofilm cells (SMICs).

|  | SMIC_50_ (mg/L) for biofilm cells^a^ | | | SMIC_90_ (mg/L) for biofilm cells^a^ | | |
| --- | --- | --- | --- | --- | --- | --- |
|  | CPFG | MCFG | AMPH-B | CPFG | MCFG | AMPH-B |
| *C. albicans* (SC5314) | <0.01 | <0.01 | 0.06 | >16 | 16 | 2 |
| *C. auris* (NCPF8985) | >16 | >16 | 1 | >16 | >16 | 2 |

^a^SMICs for biofilm cells were determined using the XTT assay.

CPFG, caspofungin; MCFG, micafungin; AMPH-B, amphotericin B.

**Table S3.** Association of intracellular accumulation of FITC-caspofungin with cell viability

|  | Cells with intracellular FITC-CPFG (%)^a^ | MB-stained dead cells (%)^b^ | Viable colony count  (× 10^4^ CFU/mL) |
| --- | --- | --- | --- |
| No drug control | 0 | 0 | 244 |
| Caspofungin in |  |  |  |
| RPMI-1640 | 1.0 ± 1.3 | 0.25 ± 0.8 | 252 |
| 0.9% NaCl | 0 | 0 | 293 |
| 0.9% KCl | 0 | 0 | 320 |
| H_2_O | 97.5 ± 3.1 | 92.8 ± 3.0 | 19.6 |
| 5% glucose | 99 ± 1.3 | 96.3 ± 3.2 | 0.96 |

*C. glabrata* cells were treated with FITC-caspofungin dissolved in different solutions for 30 min. Percentage represent the average of calculated cells ± SD.

^a^Percentage of cells with intracellular accumulation of FITC-caspofungin (FITC-CPFG)

^b^Percentage of dead cells stained with Methylene Blue (MB)

**Supplementary Methods**

**NMR spectra analysis and density functional theory calculations.** Caspofungin, micafungin, and anidulafungin (10 mg) were dissolved in 700 μL dH_2_O and transferred to NMR tubes (final drug concentration: 14 g/L). A dilution series of 1 M NaCl, KCl, and Na_2_SO_4_ (final concentrations: 28 mM, 125 mM, and 417 mM) was added to each solution. NMR spectra were recorded using a Varian 500PS NMR spectrometer (Varian Medical Systems Inc., Palo Alto, CA, USA). ^1^H and ^13^C spectra were recorded as chemical shifts (δ) in ppm relative to the solvent peak using tetramethylsilane(2,2,3,3-d4) and trimethylsilyl-3-propanoic acid sodium salt (Thermo Fisher Scientific) as internal standards. Chemical shifts (δ) are presented in ppm and coupling constants (*J*) were measured in hertz (Hz). NMR spectra were processed using an ACD/SpecManager Enterprise platform (ACD/Labs, Toronto, Canada). Caspofungin acetate was purchased from Funakoshi. Co., Ltd. (Tokyo, Japan). Density functional theory calculations were performed using Molecular Operating Environment version 2014.09 software (MOLSIS, Inc., Montreal, Canada). Initial structures were generated and optimized with the Molecular Orbital PACkage (http://openmopac.net/). The geometries were further optimized with FMO-HF/6-31G.

**Preparation and fluorescence imaging of FITC-caspofungin and viability studies.** FITC-caspofungin was prepared by the condensation of caspofungin and FITC in an apparatus with magnetic stirring in an inert atmosphere. The crude was purified by C18-reverse phase silica gel chromatography (Eluting: MeCN:MeOH:AcOH=100:100:0.2). Flash column chromatography was performed over Fuji Silysia Chemical Ltd. silica gel C60 (50-200 μm) (Aichi, Japan). Thin-layer chromatography was performed using C-18 TLC silica gel 60 F254 aluminum sheets (Merck). The product, acetate salt, was obtained in 82% yield and analyzed by mass spectroscopy on JEOL JMS-T100TD; MS (ESI) m/z Calcd for C_73_H_99_N_11_O_20_S [M+Na]^+^ 1504.6686 was 1504.787. FITC-caspofungin was dissolved in 100% DMSO at 2 mM concentration and stored at 4°C until use.

For fluorescence imaging, 1‒5 × 10^6^ CFU/mL of *C. glabrata* and MRSA cells were exposed to FITC-caspofungin dissolved in RPMI-1640, 0.9% NaCl, 0.9% KCl, dH_2_O, and 5% glucose water, respectively, for 30 min and observed under a fluorescent microscope. Keyence BZ-X700 series digital microscope (Keyence, Osaka, Japan) was used for general observation with excitation wavelength at 470 nm and emission wavelength at 520 nm with 1000X magnification. Nikon A1Rsi series confocal laser microscope (Nikon, Tokyo, Japan) was used for analyzing intracellular drug distribution with excitation wavelength at 488 nm and emission wavelength at 525 nm. Viability studies for FITC-caspofungin were performed at the concentration of 50 μM and treatment time of 30 min. Viability staining was performed with methylene blue solution where stained cells were considered dead and unstained cells were considered alive. A total of 400 cells (40 cells at ten different fields of view) were observed per group and the average of stained cells was calculated (± SD).

**Measurement of intracellular ROS levels.** The intracellular ROS level in *C. glabrata* was determined by measuring H_2_DCFDA conversion to fluorescent dichloroluorescein (DCF) as described previously with minor modifications. Briefly, 1‒5 × 10^7^ CFU/mL of *C. glabrata* cells were incubated with 50 μM of H_2_DCFDA for 30 min and exposed to caspofungin dissolved in various solutions. Fluorescence intensity (FI) was measured with PHERAstar FS plate reader (BMG LABTECH, Ortenberg, Germany) at 15 min, 30 min, and 60 min. Control groups were cell solutions that did not contain caspofungin and RFI was calculated as caspofungin treated FI/control FI. Fluorescence intensity was measured with excitation at 485 nm and emission at 520 nm (software version: 3.10R3, filmware version: 1.13, flashes per well: 10 flashes, gain: 1527, focal height: 7.3 mm, positioning delay: 0.1 second). Quintuplicate samples were analyzed for each group, and experiments were independently performed three times.
